# Supplementary material for: Viroscope: Plant viral diagnosis from high-throughput sequencing data using biologically-informed genome assembly coverage
Source: Front Microbiol. 2022 Oct 21;13:967021. doi: 10.3389/fmicb.2022.967021 (PMC9634423; doi:10.3389/fmicb.2022.967021)
Supplement: Supplementary file 1 [file Data_Sheet_1.PDF]

Supplementary Figures

Figure S1. Read assignment with HTS data from field samples (full version).
From sequencing data for field samples of cherry plants at the shoot development stage (SD-L1, SD-L2, SD-S1, and SD-S2), 10 subsets of randomly selected reads were built at different depths of sequencing. Three bioinformatic algorithms were tested, namely *Centrifuge*, *Kraken2*, and *Minimap2*. All the dots represent the average of 10 measures. Read assignment at different depths of sequencing (note the different scales of the ordinates) using the Pavium panel-I (11 viruses). Average values (dots) as well as standard deviations are listed in Supplementary Table S11. Abbreviations: SD, Shoot Development Stage; ACLSV, apple chlorotic leaf spot virus; ApMV, apple mosaic virus; CGRMV, cherry green ring mottle virus; CNRMV, cherry necrotic rusty mottle virus; CVA, cherry virus A; LChV-1, little cherry virus 1; LChV-2, little cherry virus 2; PBNSPaV, plum bark necrosis stem pitting associated virus; PDV, prune dwarf virus; PNRSV, prunus necrotic ringspot virus; PPV, plum pox virus

Figure S2. Viral genome assembly coverage with HTS data from field samples (full version).
From sequencing data for field samples of cherry plants at the shoot development stage (SD-L1, SD-L2, SD-S1, and SD-S2), 10 subsets of randomly selected reads were built at different depths of sequencing. Three bioinformatic algorithms were tested, namely *Centrifuge*, *Kraken2*, and *Minimap2*. All the dots represent the average of 10 measures. VGAC was calculated according to Materials and Methods and with the reads assigned by the different algorithms at the respective depth of sequencing. VGAC obtained from the assembly of assigned reads (ranges from 0 to 1). The cases for the 11 viruses from Pavium panel-I are presented. Average values (dots) as well as standard deviations are listed in Supplementary Table S11. Abbreviations: SD, Shoot Development Stage; VGAC, Viral Genome Assembly Coverage; ACLSV, apple chlorotic leaf spot virus; ApMV, apple mosaic virus; CGRMV, cherry green ring mottle virus; CNRMV, cherry necrotic rusty mottle virus; CVA, cherry virus A; LChV-1, little cherry virus 1; LChV-2, little cherry virus 2; PBNSPaV, plum bark necrosis stem pitting associated virus; PDV, prune dwarf virus; PNRSV, prunus necrotic ringspot virus; PPV, plum pox virus.

Figure S3. Read assignment and coverage in synthetic data (full version).
Synthetic HTS data were generated to simulate actual samples. The Pavium panel-I comprising 11 viral genomes was used to build samples containing 3% of viral reads from a 20 million paired-end reads. Samples were built with both a homogeneous distribution of viral reads (*synhom*) and based on an average distribution of actual samples (*synab*) (Supplementary Table S2 and Supplementary Table S3). In all cases, 10 subsets of randomly selected reads were built at different depths of sequencing. Assigned reads are shown in the two first column charts and VGAC is shown in the last two column charts. Average values (dots) as well as standard deviations are listed in Supplementary Table S11. Abbreviations: VGAC, Viral Genome Assembly Coverage; ACLSV, apple chlorotic leaf spot virus; ApMV, apple mosaic virus; CGRMV, cherry green ring mottle virus; CNRMV, cherry necrotic rusty mottle virus; CVA, cherry virus A; LChV-1, little cherry virus 1; LChV-2, little cherry virus 2; PBNSPaV, plum bark necrosis stem pitting associated virus; PDV, prune dwarf virus; PNRSV, prunus necrotic ringspot virus; PPV, plum pox virus.

Figure S4. Simulated mutations in synthetic HTS data (full version).
Mutated virus genomes were simulated to generate synthetic HTS dataset to evaluate read assignment tolerance to viral mutated variants. Each viral genome of the Pavium panel-I was randomly mutated at the different rates indicated (5, 10, 15, 20, 25 and 30%) at the far right of each chart. Albeit 20 million reads were generated for each mutation rate, a 10x subsampling of 10 million reads was performed, so dots represent a mean number of assigned reads. The distribution of viral reads in this case was homogeneous. Dotted red line: expected number of assigned (mapped) reads according to the distribution of reads. Average values (dots) as well as standard deviations are listed in Supplementary Table S11. Abbreviations: VGAC, Viral Genome Assembly Coverage; ACLSV, apple chlorotic leaf spot virus; ApMV, apple mosaic virus; CGRMV, cherry green ring mottle virus; CNRMV, cherry necrotic rusty mottle virus; CVA, cherry virus A; LChV-1, little cherry virus 1; LChV-2, little cherry virus 2; PBNSPaV,

plum bark necrosis stem pitting associated virus; PDV, prune dwarf virus; PNRSV, prunus necrotic ringspot virus; PPV, plum pox virus.

Figure S5. Examples of mapped reads onto viral genomes.
A. Representation of ASGV genome (6,496 bp length). **B**. Assigned reads mapped onto ASGV (dataset R2), showing they were unable to assemble a contiguous contig to contain a replicase. **C**. Representation of segment 2 (2,046 bp length) of PNRSV genome. **D**. Assigned reads mapped onto segment 2 of PNRSV (sample SS-L2), where reads were concentrated almost only in the 5'-region. In this case, there were no reads mapping onto the other two segments (replicase is contained in segment 1). Abbreviations: ASGV, apple stem grooving virus; PNRSV, prunus necrotic ringspot virus; SS, Senescense Stage.

Figure S6. Viral pathogen detection in different infected plants of *Prunus sp.* according to the panel.
A two-step singleplex RT-PCR analysis for the 11 viruses (lane 1–11) and the internal control (lane 12) was performed using specific primers. Leaf samples: (A) SS-L1; (B) SS-L2; (C) SS-S1; (D) SS-S2. The specific amplification products of the 11 viral pathogens (E) and the corresponding non-template control (NTC) for each RT-PCR reaction (F) are shown. Lane 1: ACLSV; lane 2: ApMV; lane 3: CGRMV; lane 4: CNRMV; lane 5: CVA; lane 6: LChV-1; lane 7: LChV-2; lane 8: PBNSPaV; lane 9: PDV; lane 10: PNRSV; lane 11: PPV and lane 12: PDS-1. MM: 100 bp molecular weight marker. The non-specific bands observed in sample S1 (C; lane 3) are not interfering with the expected PCR fragment for the virus CGRMV which is 181 bp long. Abbreviations: ACLSV, apple chlorotic leaf spot virus; ApMV, apple mosaic virus; CGRMV, cherry green ring mottle virus; CNRMV, cherry necrotic rusty mottle virus; CVA, cherry virus A; LChV-1, little cherry virus 1; LChV-2, little cherry virus 2; PBNSPaV, plum bark necrosis stem pitting associated virus; PDV, prune dwarf virus; PNRSV, prunus necrotic ringspot virus; PPV, plum pox virus; SS, Senescence Stage.

Figure S7. Diagnosis on field samples from the shoot development stage using Pavium panel-II.

HTS data from field samples (SD-L1, SD-L2, SD-S1, and SD-S2) were submitted to the pipeline using the Pavium panel-II. Left ordinate: number of viral reads (different scales); right ordinate: VGAC scale; circles: VGAC values for each software; filled circles: replicase identified; dotted lines: VGAC cutoffs at 0.1 and 0.3. Abbreviations: SD, Shoot Development Stage; VGAC, Viral Genome Assembly Coverage; ACLSV, apple chlorotic leaf spot virus; ApMV, apple mosaic virus; CGRMV, cherry green ring mottle virus; CNRMV, cherry necrotic rusty mottle virus; CVA, cherry virus A; LChV-1, little cherry virus 1; LChV-2, little cherry virus 2; PBNSPaV, plum bark necrosis stem pitting associated virus; PDV, prune dwarf virus; PNRSV, prunus necrotic ringspot virus; PPV, plum pox virus.

Figure S8. Diagnosis on field samples from the senescence stage using Pavium panel-II.

HTS data from field samples (SS-L1, SS-L2, SS-S1, and SS-S2) were submitted to the pipeline using the Pavium panel-II. Left ordinate: number of viral reads (different scales); right ordinate: VGAC scale; circles: VGAC values for each software; filled circles: replicase identified; dotted lines: VGAC cutoffs at 0.1 and 0.3. Abbreviations: SS, Senescence Stage; VGAC, Viral Genome Assembly Coverage; ACLSV, apple chlorotic leaf spot virus; ApMV, apple mosaic virus; CGRMV, cherry green ring mottle virus; CNRMV, cherry necrotic rusty mottle virus; CVA, cherry virus A; LChV-1, little cherry virus 1; LChV-2, little cherry virus 2; PBNSPaV, plum bark necrosis stem pitting associated virus; PDV, prune dwarf virus; PNRSV, prunus necrotic ringspot virus; PPV, plum pox virus.

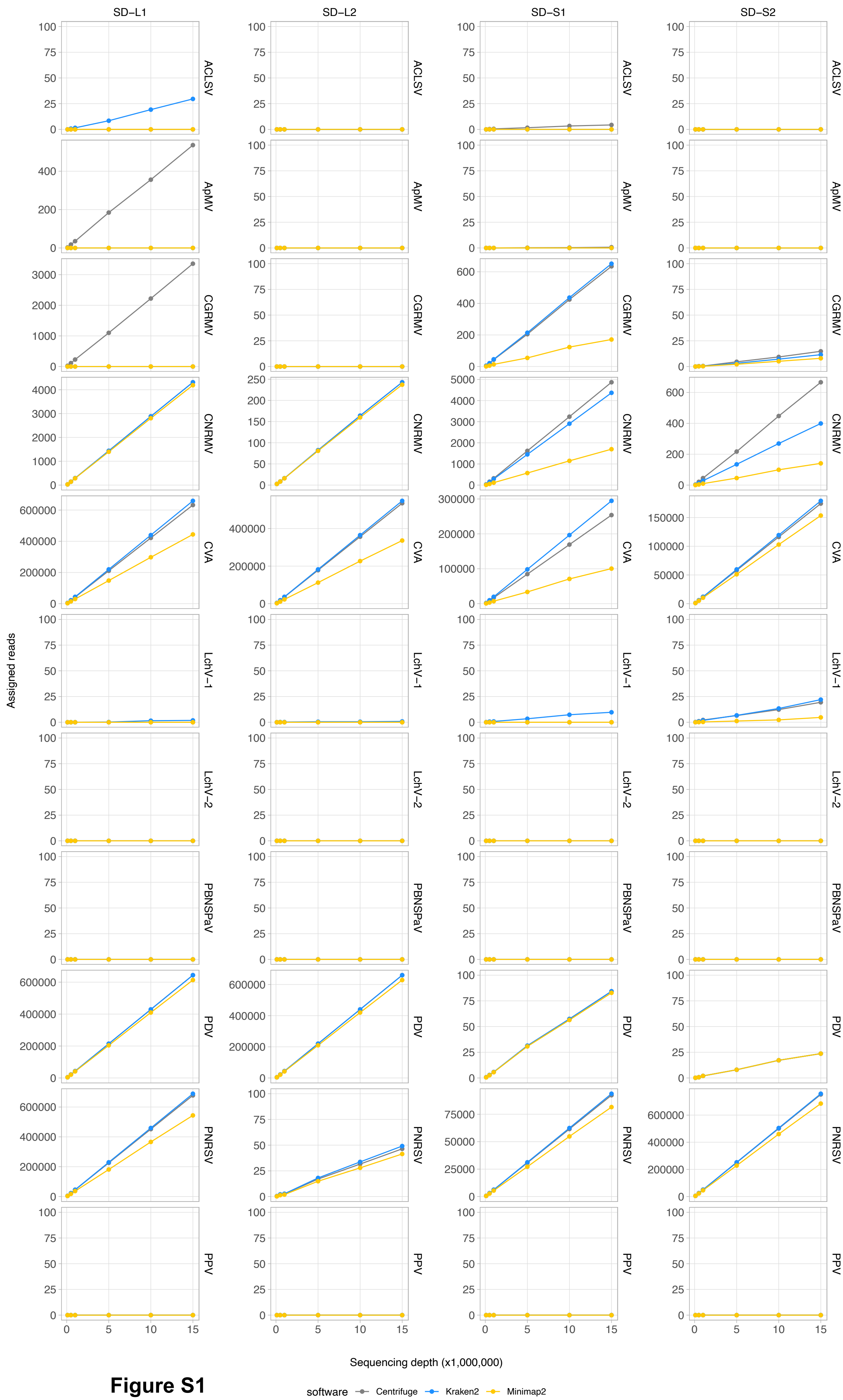
SD−L1
SD−L2
SD−S1
SD−S2
ACLSV
ApMV
CGRMV
CNRMV
CVA
LchV−1
LchV−2
PBNSPaV
PDV
PNRSV
PPV
Assigned reads
Sequencing depth (x1,000,000)
software
Centrifuge
Kraken2
Minimap2

Figure S1

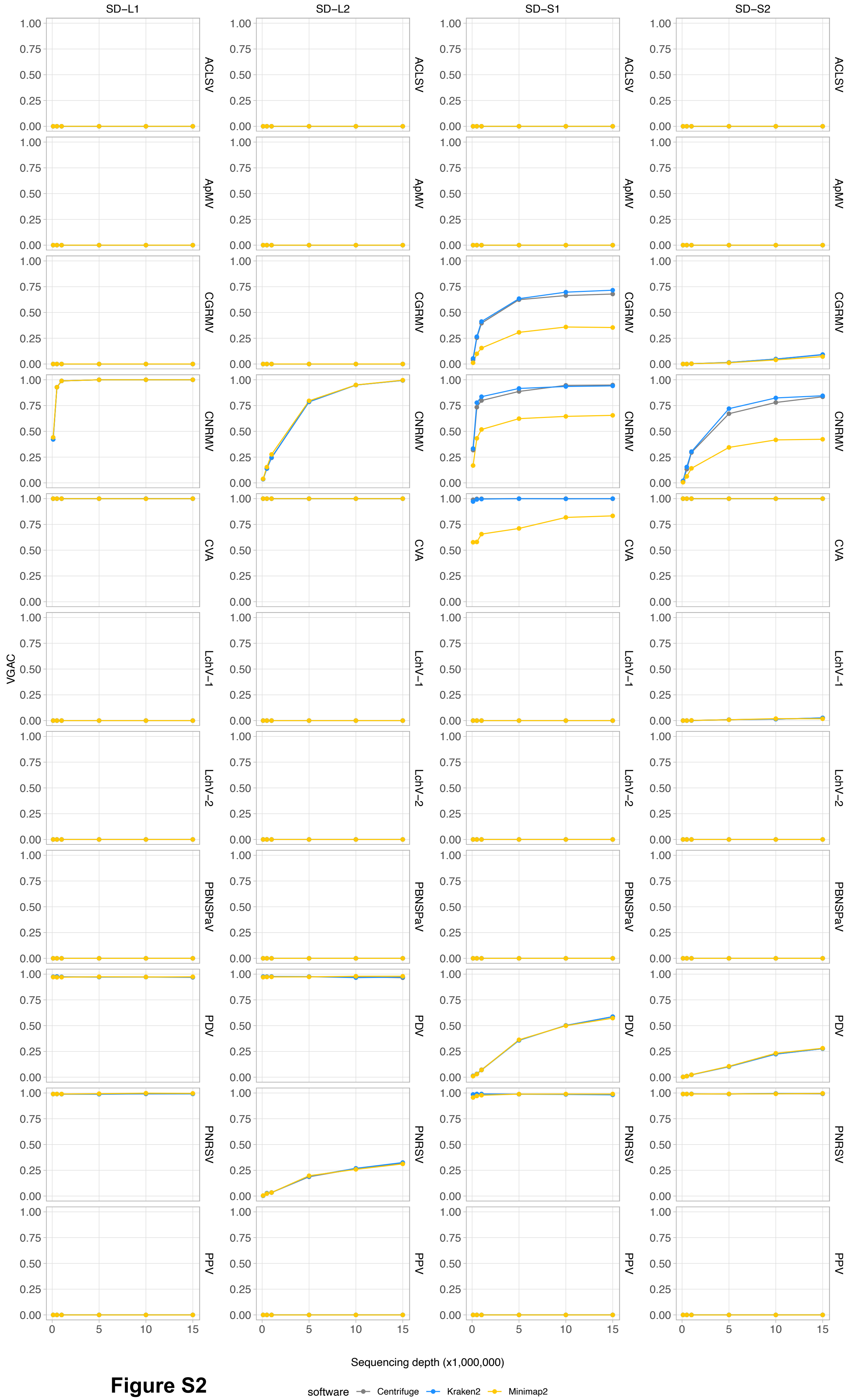
SD–L1
SD–L2
SD–S1
SD–S2
ACLSV
ApMV
CGRMV
CNRMV
CVA
LchV–1
LchV–2
PBNSPaV
PDV
PNRSV
PPV
VGAC
Sequencing depth (x1,000,000)
software
Centrifuge
Kraken2
Minimap2

Figure S2

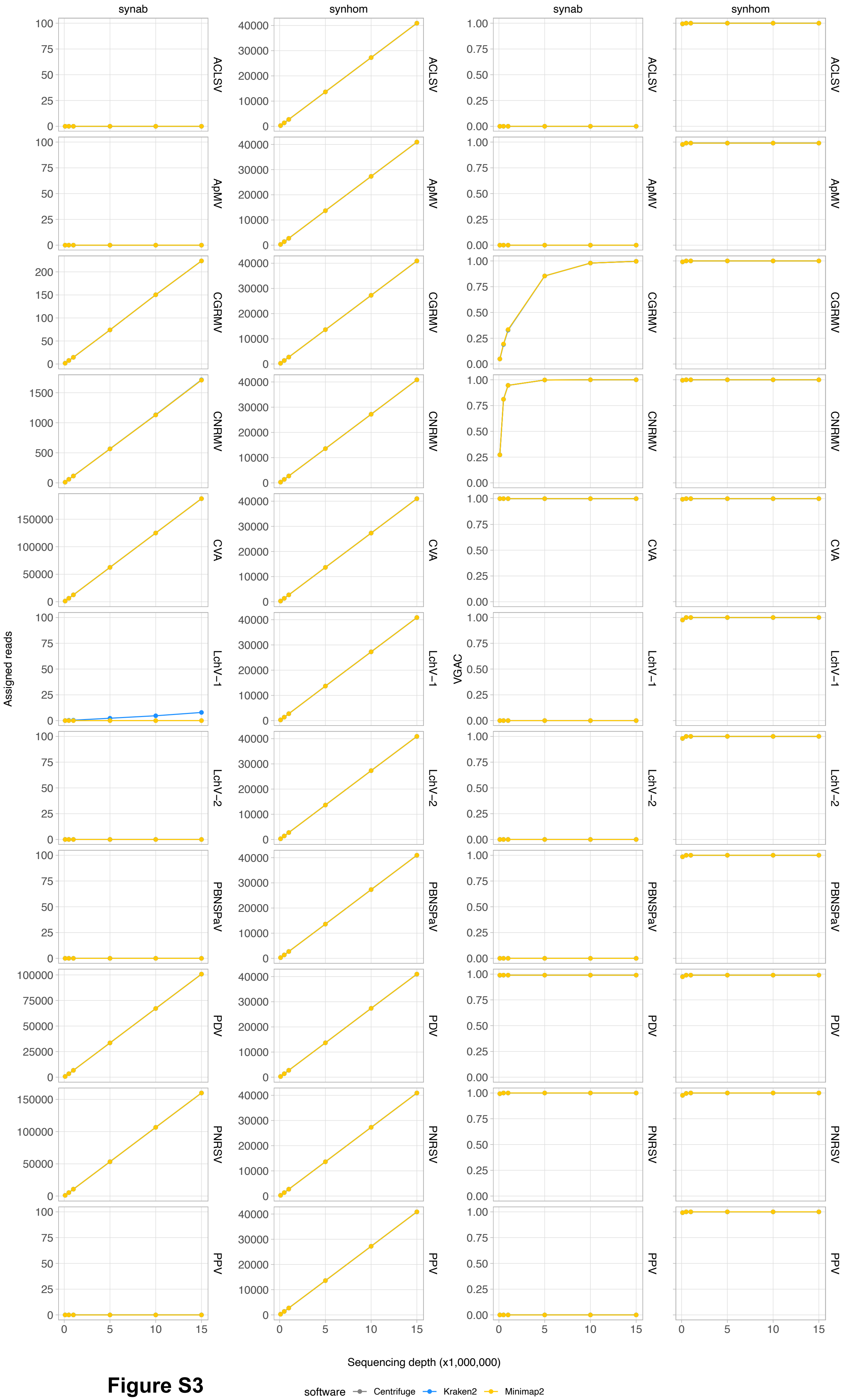
synab
synhom
synab
synhom
ACLSV
ApMV
CGRMV
CNRMV
CVA
LchV−1
LchV−2
PBNSPaV
PDV
PNRSV
PPV
Assigned reads
VGAC
Sequencing depth (x1,000,000)
software
Centrifuge
Kraken2
Minimap2

Figure S3

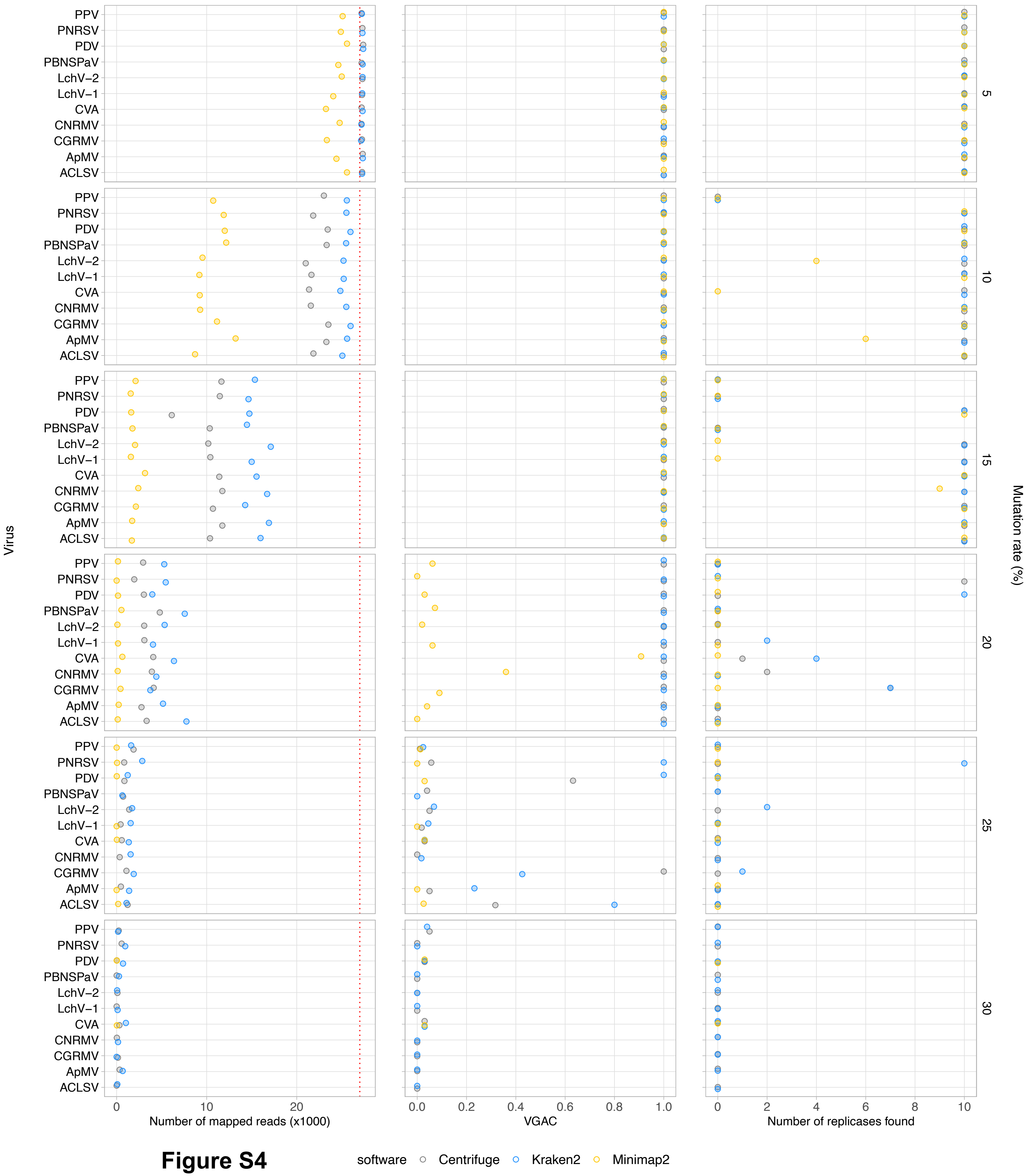
Virus
PPV
PNRSV
PDV
PBNSPaV
LchV−2
LchV−1
CVA
CNRMV
CGRMV
ApMV
ACLSV
Number of mapped reads (x1000)
VGAC
Number of replicases found
Mutation rate (%)
5
10
15
20
25
30
software
Centrifuge
Kraken2
Minimap2

Figure S4

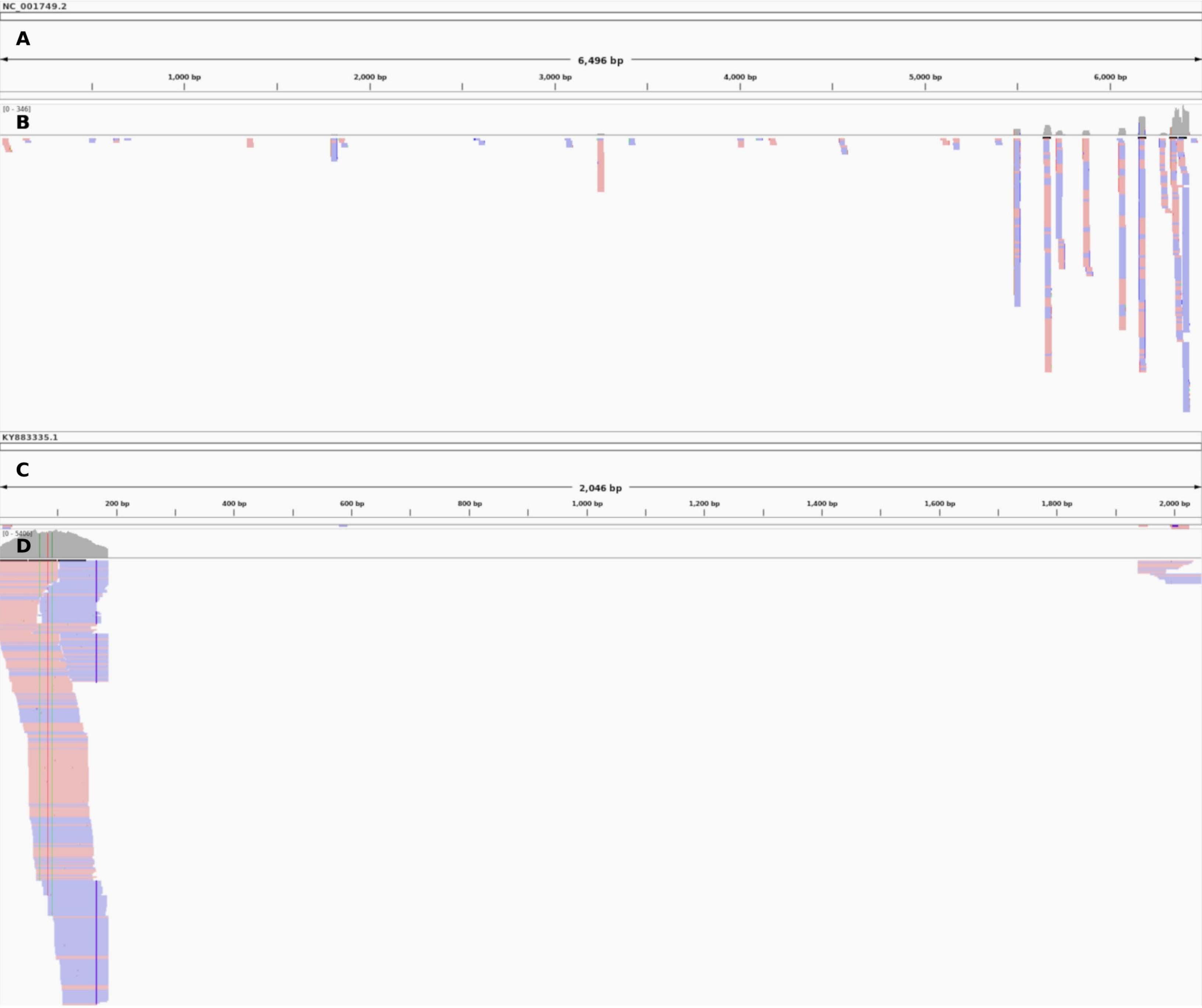
NC_001749.2
A
6,496 bp
1,000 bp
2,000 bp
3,000 bp
4,000 bp
5,000 bp
6,000 bp
[0 - 346]
B
KY883335.1
C
2,046 bp
200 bp
400 bp
600 bp
800 bp
1,000 bp
1,200 bp
1,400 bp
1,600 bp
1,800 bp
2,000 bp
D

Figure S5

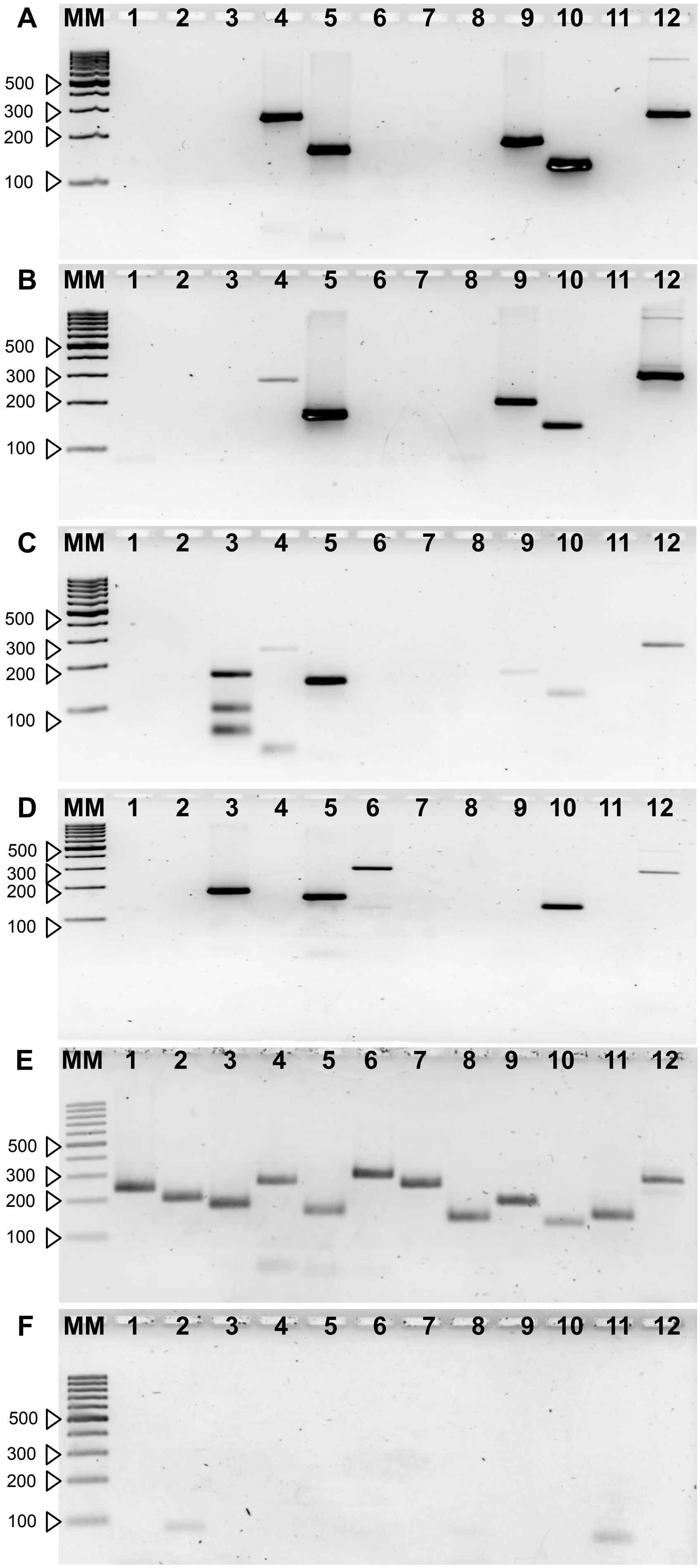
A MM 1 2 3 4 5 6 7 8 9 10 11 12
500
300
200
100
B MM 1 2 3 4 5 6 7 8 9 10 11 12
500
300
200
100
C MM 1 2 3 4 5 6 7 8 9 10 11 12
500
300
200
100
D MM 1 2 3 4 5 6 7 8 9 10 11 12
500
300
200
100
E MM 1 2 3 4 5 6 7 8 9 10 11 12
500
300
200
100
F MM 1 2 3 4 5 6 7 8 9 10 11 12
500
300
200
100

Figure S6

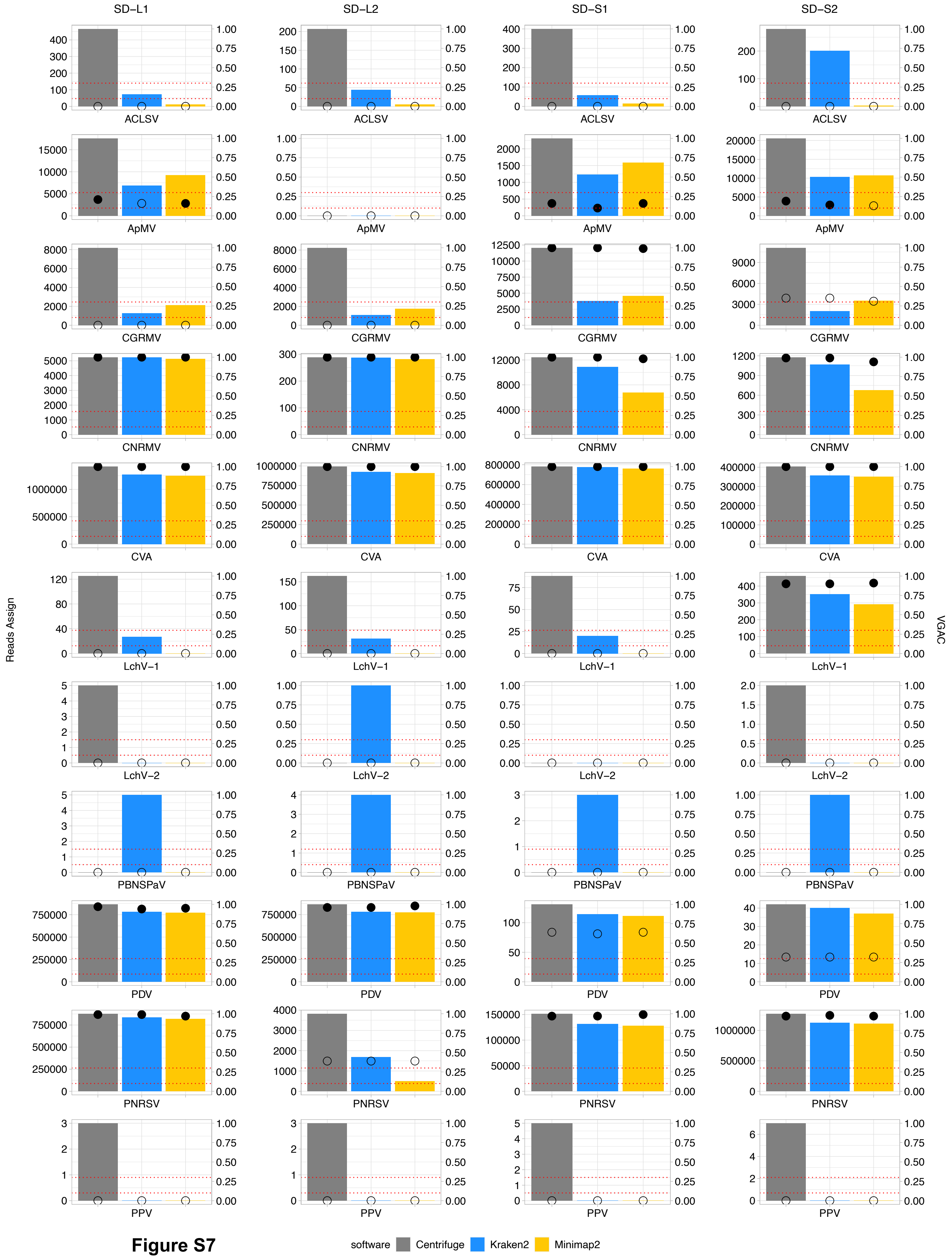
SD−L1
SD−L2
SD−S1
SD−S2
Reads Assign
VGAC
ACLSV
ApMV
CGRMV
CNRMV
CVA
LchV−1
LchV−2
PBNSPaV
PDV
PNRSV
PPV
software
Centrifuge
Kraken2
Minimap2

Figure S7

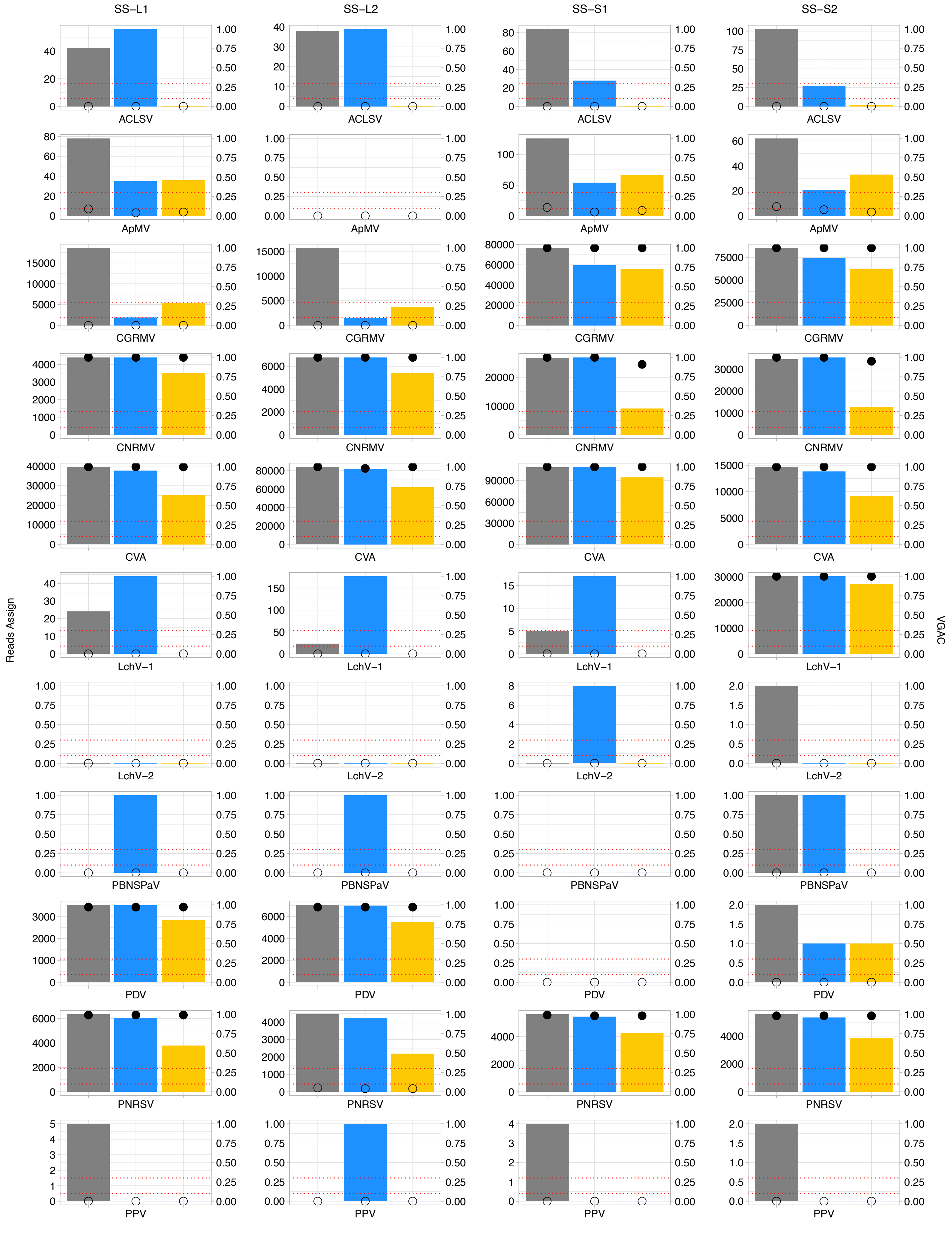

SS–L1
SS–L2
SS–S1
SS–S2
ACLSV
ApMV
CGRMV
CNRMV
CVA
LchV–1
LchV–2
PBNSPaV
PDV
PNRSV
PPV
Reads Assign
VGAC
software
Centrifuge
Kraken2
Minimap2

Figure S8
